# Endoplasmic reticulum stress: major player in size-dependent inhibition of P-glycoprotein by silver nanoparticles in multidrug-resistant breast cancer cells

**DOI:** 10.1186/s12951-019-0448-4

**Published:** 2019-01-22

**Authors:** Mohana Krishna Gopisetty, Dávid Kovács, Nóra Igaz, Andrea Rónavári, Péter Bélteky, Zsolt Rázga, Viktória Venglovecz, Bálint Csoboz, Imre Miklós Boros, Zoltán Kónya, Mónika Kiricsi

**Affiliations:** 1 Department of Biochemistry and Molecular Biology, Faculty of Science and Informatics, University of Szeged, Közép fasor 52, Szeged, 6726 Hungary; 2 Department of Applied and Environmental Chemistry, University of Szeged, Rerrich B. tér 1, Szeged, 6720 Hungary; 3 Department of Pathology, University of Szeged, Állomás u. 2, Szeged, 6725 Hungary; 4 Department of Pharmacology and Pharmacotherapy, University of Szeged, Dóm tér 12, Szeged, 6720 Hungary; 5 Institute of Biochemistry, Biological Research Center of the Hungarian Academy of Sciences, Temesvári krt. 62, Szeged, 6726 Hungary; 6 MTA-SZTE Reaction Kinetics and Surface Chemistry Research Group, Rerrich B. tér 1, Szeged, 6720 Hungary

**Keywords:** Silver nanoparticles, Multidrug resistance, P-glycoprotein, ER stress

## Abstract

**Background:**

Development of multidrug resistance (MDR) is a major burden of successful chemotherapy, therefore, novel approaches to defeat MDR are imperative. Although the remarkable anti-cancer propensity of silver nanoparticles (AgNP) has been demonstrated and their potential application in MDR cancer has been proposed, the nanoparticle size-dependent cellular events directing P-glycoprotein (Pgp) expression and activity in MDR cancer have never been addressed. Hence, in the present study we examined AgNP size-dependent cellular features in multidrug resistant breast cancer cells.

**Results:**

In this study we report that 75 nm AgNPs inhibited significantly Pgp efflux activity in drug-resistant breast cancer cells and potentiated the apoptotic effect of doxorubicin, which features were not observed upon 5 nm AgNP treatment. Although both sized AgNPs induced significant ROS production and mitochondrial damage, 5 nm AgNPs were more potent than 75 nm AgNPs in this respect, therefore, these effects can not to be accounted for the reduced transport activity of ATP-driven pumps observed after 75 nm AgNP treatments. Instead we found that 75 nm AgNPs depleted endoplasmic reticulum (ER) calcium stores, caused notable ER stress and decreased plasma membrane positioning of Pgp.

**Conclusion:**

Our study suggests that AgNPs are potent inhibitors of Pgp function and are promising agents for sensitizing multidrug resistant breast cancers to anticancer drugs. This potency is determined by their size, since 75 nm AgNPs are more efficient than smaller counterparts. This is a highly relevant finding as it renders AgNPs attractive candidates in rational design of therapeutically useful agents for tumor targeting. In the present study we provide evidence that exploitation of ER stress can be a propitious target in defeating multidrug resistance in cancers.

**Electronic supplementary material:**

The online version of this article (10.1186/s12951-019-0448-4) contains supplementary material, which is available to authorized users.

## Background

Every year 1.38 million women are diagnosed with breast cancer [1]. Although breast cancer is generally considered fairly chemo-responsive, it can attain resistance to a large number of anti-cancer drugs. This multidrug-resistant (MDR) phenotype exhibits many distinctive cellular features such as increased tolerance to oxidative stress and apoptosis, activated DNA repair and modulated signal transduction pathways [2, 3], however, the principal component of MDR is linked to P-glycoprotein (Pgp) overexpression [4, 5]. As a result, various structurally and functionally unrelated chemotherapy agents are actively exported via ATP-fuelled efflux pumps [6], creating a severe impediment to successful chemotherapy. Pgp is the most studied member of the ATP-binding cassette-type membrane transporters and has gained interest due to its ubiquitous nature and owing to its central position as a point of convergence of several deregulated molecular mechanisms [7], such as the PI3K/AKT pathway [8], NF-kB pathway [8, 9] and epigenetic regulations [10, 11] in drug-resistant cancers. Inhibition of Pgp activity could be the ultimate solution to improve the success rate of conventional chemotherapy. Although significant scientific effort focused on the development of Pgp inhibitors, most of them were dismissed on the grounds of safety, efficacy and disappointing performance in clinical trials [12].

Silver nanoparticles (AgNPs) have been extensively studied in recent years, thus their unique physicochemical, antibacterial, antifungal and antiviral features are already characterized in detail, however, at the same time it was suggested that AgNPs might have a potential in cancer therapy owing to their prominent anti-proliferative and cytotoxic features [13, 14]. In fact, it has been demonstrated that AgNPs trigger the generation of reactive oxygen species (ROS), unbalance the cellular redox homeostasis, induce cell cycle arrest and decrease cancer cell viability in vitro and inhibit the growth of tumor tissues in vivo [15–22]. We have verified that AgNPs target mitochondria and induce tumor suppressor p53-independent cancer cell apoptosis [23]. Apart from modulating regulatory pathways leading to apoptotic cell death [24, 25], exposure to AgNPs might lead to the accumulation and aggregation of misfolded proteins, activation of endoplasmic reticulum (ER) stress and unfolded protein response (UPR) [26]. All these cellular mechanisms related to AgNP-induced stress response might depend on the actual physical and chemical properties of nanoparticles, like the nature of the capping material, as well as nanoparticle size and shape.

We have recently demonstrated, that AgNPs of 28 nm diameter are able to modulate the drug efflux activity and enhance chemotherapy in multidrug-resistant colon cancer cells [27]. An in vivo study on an MDR cancer model also revealed enhanced antitumor effects of 8 nm sized AgNPs functionalized with cell penetrating peptides [28]. These data suggest that AgNPs are able to target the MDR-related biological profile of tumor cells, but the molecular background of the reduced transport activity and its dependence on the nanoparticle diameters remain elusive. Therefore, our main goal was to investigate whether the actual AgNP size would influence the AgNP-induced molecular mechanisms and the inhibitory actions on P-glycoprotein in multidrug-resistant breast cancer cells. For this, quasi-spherical citrate-coated silver nanoparticles of two different sizes (5 nm and 75 nm diameter) were synthetized and the cellular events underlying Pgp inhibition were studied in drug-sensitive MCF-7 and drug-resistant MCF-7/KCR breast adenocarcinoma cells.

## Methods

### Cell culture

The MCF-7 human breast adenocarcinoma cell line was purchased from ATCC. The drug-resistant MCF-7/KCR cell line was developed from MCF-7 under doxorubicin selection pressure from 10 nM to 1 µM [29, 30]. Cell lines were maintained, and treatments were applied in RPMI-1640 (LONZA) medium supplemented with 10% FBS, 2 mM glutamine and penicillin–streptomycin solution at 37 °C, 5% CO_2_ and 95% humidity. To maintain the drug-resistant phenotype, MCF-7/KCR cells were cultured in media with and without 1 µM doxorubicin for 1 week each. Before experiments, MCF-7/KCR cells were grown in doxorubicin-free medium.

### Synthesis and characterization of AgNPs

Citrate-capped silver nanoparticles were synthesized according to Wan et al. with modifications [31]. Briefly, to obtain 5 nm AgNPs, 75 mL water and 20 mL 1% citrate solution were mixed and heated, then 1.7 mL of 1% AgNO_3_ solution and 2 mL of 0.1% NaBH_4_ solution were added under vigorous stirring at 70 °C. The resulting AgNPs were used as starter seeds for larger AgNPs in a stepwise growth approach by adding 2 mL of 1% citrate solution, 75 mL water and 2 mL of 1% AgNO_3_ in three subsequent cycles.

Morphology and size distribution of the synthetized nanoparticles was characterized by transmission electron microscopy using FEI Tecnai G2 20× microscope at 200 kV acceleration voltage and by Dynamic Light Scattering using Malvern Zetasizer Nano instrument.

### Rhodamine 123 accumulation assay

Cells (2 × 10^6^ cells/well) were treated with 5 nm or 75 nm AgNPs in 150 µM concentration for 65 h or with verapamil in 40 µM concentration for 2 h. Then cells were washed and resuspended in serum-free RPMI-1640 medium containing 10 µM of Rhodamine 123 (RH123, Sigma-Aldrich). Following 2 h incubation, cells were washed and RH123 fluorescence of at least 10,000 cells/sample were measured by flow cytometry using FACSCalibur™ platform. Data were analyzed by FlowJo V10 software. Results were obtained from three independent experiments.

### Preparation of plasma membrane and cytoplasmic fractions

To obtain plasma membrane and cytoplasmic fractions [32], MCF-7/KCR cells (2 × 10^6^ cells/dish) were collected in ice cold TNM buffer (10 mM NaCl, 1.5 mM MgCl_2_, 10 mM Tris–HCl pH 7.4) and homogenized using glass beads (Sigma). Lysates were centrifuged at 2000*g*, the supernatant was collected and centrifuged at 8000*g* at 4 °C using Sorvall-RC-28S centrifuge. Supernatant was considered as cytoplasmic fraction. The pellet was resuspended in 1 mL ice cold TNM buffer and was layered on TNM buffer containing 36% sucrose. Samples were centrifuged (Sorvall-WX-Ultra80) at 100,000*g*, at 4 °C overnight. The interphase was collected and subjected to protein precipitation using trichloroacetic acid. After centrifugation at 18,000*g*, the pellet was washed with acetone and dissolved in 2×Laemmli Buffer (130 mM TrisHCl pH 6.8, 10% ß-mercaptoethanol, 4% SDS, 20% glycerin, 0.01% bromophenol blue), which was considered as plasma membrane fraction.

### Immunoblotting

Whole cell extracts were prepared using RIPA lysis buffer (50 mM Tris (pH:7.4), 150 mM NaCl, 1 mM EDTA, 1% Triton X-100 and 1xPIC). To detect cytoplasmic cytochrome c, cells were lysed in sonication buffer (50 mM Tris, 2 mM EDTA, 0.5 mM DTT, 50 mM NaCl, 1xPIC), centrifuged at 13,000 rpm and supernatants were collected. 25 µg protein from whole cell lysates, cytoplasmic or plasma membrane fractions were resolved on 10% SDS-PAGE and transferred to nitrocellulose membrane (Amersham). Membranes were blocked with 5% non-fat dry milk in TBST (20 mM Tris, 150 mM NaCl and 0.05% Tween20). Membranes were incubated overnight with primary antibodies (Table 1) diluted in TBST containing 1% non-fat dry milk. Then species-specific HRP-conjugated secondary antibodies (DAKO) were applied. Membranes were developed with ECL reagent (Millipore) and visualized by C-DiGit Blot Scanner (LI-COR). Densitometry was performed using ImageJ software. The presented images are representative blots from three individual experiments.

**Table 1:** List of primary antibodies (with the appropriate dilutions) applied in western blotting and list and sequence of primers used for qRT-PCR

Antibody	Manufacturer and cat no	Dilution
Grp94	Santa Cruz sc-13119	1:1500
Grp78	Santa Cruz sc-376768	1:250
GADD153	Santa Cruz sc-7351	1:200
P-glycoprotein	Santa Cruz sc-55510	1:500
EDEM	Santa Cruz sc-377394	1:200
Cytochrome c	Abcam ab13575	1:500
LC3-A/B	Cell signaling 12741	1:2000
Na^+^/K^+^ ATPase	Santa Cruz sc-21712	1:200
α-tubulin	eBioscience 14-4502-82	1:1000

Primer	Forward	Reverse
Grp94	CAGTTTTGGATCTTGCTGT	CAGCTGTAGATTCCTTTGC
Grp78	TGTTCAACCAATTATCAGCAAACTC	TTCTGCTGTATCCTCTTCACCAGT
GADD153	GGAGCATCAGTCCCCCACTT	TGTGGGATTGAGGGTCACATC
EDEM	TTGACAAAGATTCCACCGTCC	TGTGAGCAGAAAGGAGGCTTC
GAPDH	TGCACCACCAACTGCTTAGC	GGCATGGACTGTGGTCATGAG

### Cell viability assay

Cells were seeded at 10^4^/well density in 96-well plates. On the following day cells were treated with either AgNPs or doxorubicin or their combination. Following treatments cells were washed and incubated with RPMI-1640 medium containing 0.5 mg/mL MTT reagent (Sigma-Aldrich). Formazan crystals were solubilized in DMSO and absorbance was measured at 570 nm using a Synergy HTX microplate reader (BIOTEK^®^). Measurements were repeated three times using 4 independent biological replicates. Absorbance values of the untreated control samples were considered as 100% viability.

### Apoptosis detection

Cells were seeded at 2 × 10^6^ cells/well density in 6-well plates. On the following day cells were treated either with AgNPs or doxorubicin or with verapamil or their combination. Cells were collected and Dead Cell Apoptosis Kit containing AnnexinV-FITC and propidium iodide (Life Technologies) was used according to the manufacturer’s recommendation. Fluorescence intensities of at least 10,000 cells/sample were measured by FACSCalibur™ and data were analyzed by FlowJo V10 software. Experiments were repeated three times using at least three independent biological replicates.

### JC-1 staining

To measure mitochondrial membrane potential via JC-1 staining, cells were seeded onto cover slips placed into 24-well plates (10^5^ cells/well). On the next day cells were treated with 150 µM of 5 nm or 75 nm AgNPs for 48 h or with apoptosis inducer M627 (12H-benzo{alpha}phenothiazine) [33] in 50 µg/mL concentration for 24 h. JC-1 is Pgp substrate, hence before JC-1 loading, 40 µM of verapamil was added to the samples (Additional file 1). After an hour cells were washed and incubated with RPMI-1640 medium containing 10 µg/mL JC-1 (Life Technologies) for 15 min. Cover slips were inversely mounted in Fluoromount™ (ThermoFisher) on glass slides and JC-1 fluorescence was visualized by OLYMPUS BX51 microscope equipped with Olympus DP70 camera using the same exposition time for all samples. Image analysis was performed by ImageJ software. Experiments were repeated three times using three independent biological replicates.

### Detection of ROS

ROS production upon AgNP treatments was detected by 2′,7′-dichlorofluorescein diacetate (DCFDA) staining. Cells were seeded onto gelatin-coated cover slips placed in 24-well plates, at 10^5^ cells/well density. Cells were treated with 150 µM of AgNPs for 48 h, then were incubated with RPMI-1640 containing 10 µM DCFDA (Sigma-Aldrich) in dark for 20 min. Cover slips were mounted on glass slides, and DCF fluorescence was visualized by OLYMPUS BX51 microscope equipped with Olympus DP70 camera using the same exposition time for all samples. Fluorescence intensity measurements were performed using ImageJ software. Measurements were repeated three times using three independent biological replicates.

### Reverse transcription and real-time RT-PCR

Total cellular RNA was prepared using RNeasy^®^ Mini Kit (QIAGEN) according to the manufacturer’s recommendation. Two microgram RNA was reverse transcribed (TaqMan^®^ Reverse Transcription kit, Applied Biosystems) in 50 µL total volume. PCR reactions were performed on PicoReal™ Real-time PCR (Thermo Scientific) using SYBRGreen qPCR Master Mix (Thermo Scientific) with an input of 1 µL cDNA. Each primer (Table 1) was used at 200 nM concentration. Relative transcript levels were determined by the ΔΔCt analysis using GAPDH as reference gene. Experiments were repeated three times using three biological replicates.

### Cytoplasmic calcium release measurements

MCF-7/KCR cells at 5 × 10^3^ density were seeded onto coverslips, which formed the base of a perfusion chamber. Cells were treated with 4 µM Quinidine (Pgp inhibitor, Sigma) and were preloaded with the Ca^2+^-sensitive fluorescence dye, Fluo4-AM (Sigma) at 5 µM for 20 min at 37 °C. The chamber was mounted on the stage of a Zeiss LSM880 confocal laser scanning microscope and cells were bathed with standard HEPES solution (140 mM NaCl, 5 mM KCl, 10 mM HEPES acid, 1 mM CaCl_2_, 1 mM MgCl_2_, 10 mM glucose) or with 100 µM carbachol (Sigma) in HEPES at 37 °C at 5–6 mL/min perfusion rate. All experiments were performed using a Plan-Apochromat 40X/1.4 oil immersion objective. 6–10 region of interests (ROIs) were examined in each experiment. Changes in intracellular Ca^2+^ concentration were determined by excitation at 488 nm, with emitted light monitored at 516 nm. Fluorescence signals were normalized to initial fluorescence intensity (F/F_0_) and expressed as relative fluorescence (ΔF/F_0_), where ΔF indicates the changes in fluorescence intensity and F_0_ is the baseline level.

## Results

### Silver nanoparticles induce size-dependent cytotoxicity in breast cancer cells

**Fig. 1:**
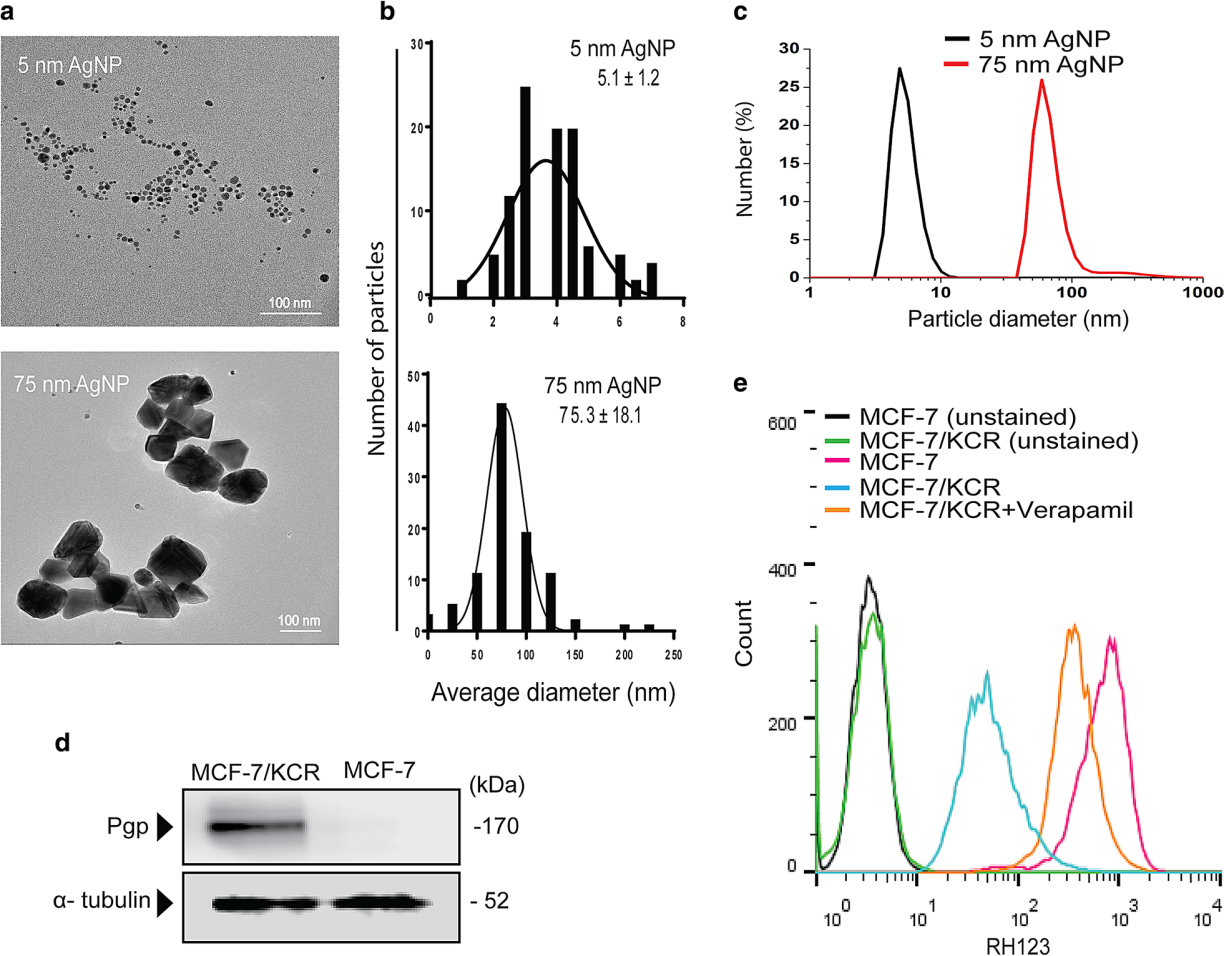
Size-dependent cytotoxicity by AgNPs in MCF-7 and drug-resistant MCF-7/KCR cells. **a** Representative TEM micrographs of the synthesized citrate-coated AgNPs. **b** Size distribution and particle diameter of AgNPs by TEM image analysis and **c** by DLS. **d** Representative Western blot of P-glycoprotein levels in MCF-7 and MCF-7/KCR cells. **e** Histogram of Rhodamine 123 (RH123) retention in MCF-7 and MCF-7/KCR cells

The successful synthesis of quasi-spherical, citrate-coated silver nanoparticles of approximately 5 nm and 75 nm in diameter was verified by transmission electron microscopy (TEM) and the respective size distributions were assessed by image analysis and by Dynamic Light Scattering (DLS) measurements (Fig. 1a–c).

AgNPs were applied on the drug-sensitive breast adenocarcinoma MCF-7 as well as on the drug-resistant MCF-7/KCR cell lines. According to TEM micrographs both cells have taken up AgNPs, 5 nm particles were localized in membrane-coated bodies while 75 nm AgNPs were found mainly in the cytoplasm of the cells (Additional file 2). No AgNPs were found in nuclei, mitochondria or within the endoplasmic reticulum.

Drug-resistant phenotype of MCF-7/KCR was verified by immunoblotting as a massive expression of P-glycoprotein was detected, while the drug-sensitive MCF-7 cells were lacking this ABC transporter (Fig. 1d). In line with this, pronounced efflux activity of the drug-resistant cells was confirmed (Fig. 1e). The Pgp inhibitor verapamil effectively inhibited the exclusion of the Pgp substrate RH123 dye indicating that the elevated efflux activity of MCF-7/KCR cells is mainly the result of Pgp overexpression (Fig. 1e).

Cytotoxicity of AgNPs on MCF-7 and MCF-7/KCR cells was determined using MTT assay. IC_50_ values (Table 2) indicated that toxicity depended on the nanoparticle size, on the treatment time, as well as on the cell type. As expected, smaller AgNPs were more cytotoxic than 75 nm counterparts to both drug-sensitive and resistant cells. Moreover, 48-h treatments were more effective on adenocarcinoma cells than 24-h exposures to either sized AgNPs. Although MCF-7 cells were more sensitive to both AgNPs, drug-resistant MCF-7/KCR cells could be also defeated efficiently by 5 nm and by 75 nm AgNPs.

**Table 2:** IC_50_ values for MCF-7 and MCF-7/KCR cells after 24 and 48-h treatments with 5 nm and 75 nm AgNPs

	24 h		48 h	
	5 nm AgNP (µM)	75 nm AgNP (µM)	5 nm AgNP (µM)	75 nm AgNP (µM)
MCF-7	212 ± 1.0	284.2 ± 1.1	179.4 ± 1.0	222.2 ± 1.1
MCF-7/KCR	244.1 ± 1.0	414.7 ± 1.2	232.9 ± 1.1	259.9 ± 1.1

### Inhibition of Pgp by 75 nm AgNPs sensitizes drug-resistant cells to doxorubicin-induced apoptosis

**Fig. 2:**
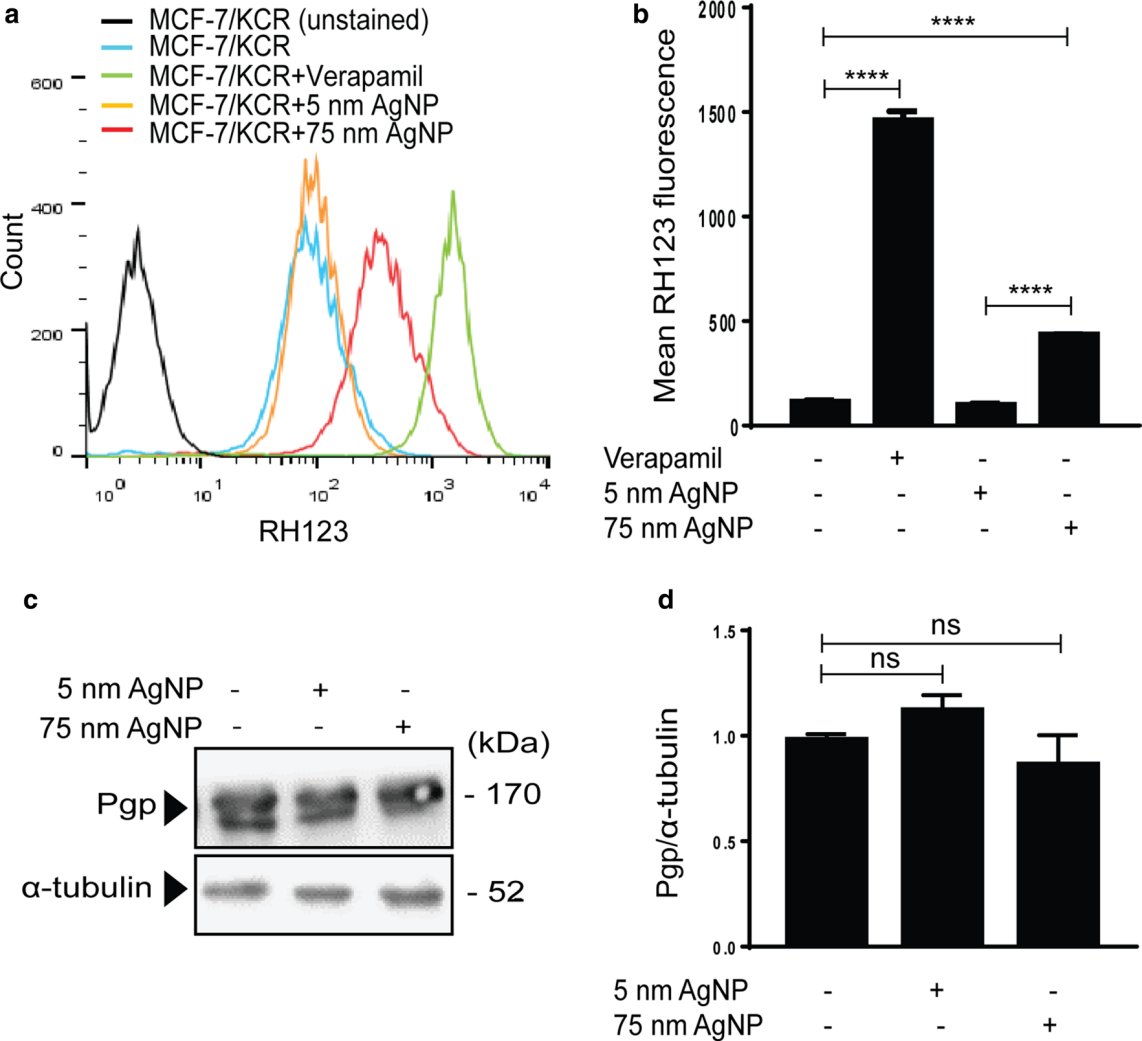
Treatments with 75 nm AgNPs reduce the efflux activity of drug-resistant MCF-7/KCR cells without causing significant changes in Pgp expression level. **a** Histograms of Rhodamine 123 accumulation and **b** mean Rhodamine 123 fluorescence of verapamil-treated, 5 nm or 75 nm AgNP-treated MCF-7/KCR cells. **c** Western blot of Pgp protein levels in MCF-7/KCR cells treated with 5 nm or 75 nm AgNPs. **d** Densitometric quantitation of Pgp western blots. Values are the means ± standard deviations of three independent experiments (****P < 0.0001, ns, non-significant, Fisher’s LSD test)

As AgNP treatments might influence Pgp efflux activity of drug-resistant cells, we exposed MCF-7/KCR cells to 5 nm or to 75 nm AgNPs and the accumulation of RH123 dye was assessed by flow cytometry (Fig. 2a, b). Administration of Pgp inhibitor verapamil resulted in high retention of RH123 in drug-resistant cells. Treatments of MCF-7/KCR cells with 5 nm AgNPs led to comparable RH123 fluorescence intensities as those of control cells. Remarkably, application of 75 nm AgNPs inhibited significantly the efflux activity of MCF-7/KCR cells (Fig. 2a, b), indicating that functional inactivation of Pgp efflux depends largely on nanoparticle size.

Attenuated Pgp protein expression may account for the inhibitory effect of 75 nm AgNPs on the efflux activity. To examine this, we determined Pgp protein levels of MCF-7/KCR cells treated with AgNPs by immunoblot. Surprisingly, we found no changes in Pgp protein levels (Fig. 2c, d). Therefore, we concluded, that the reduced transporter activity observed following 75 nm AgNP treatments is not coupled to modulated Pgp protein expression in MCF-7/KCR cells.

**Fig. 3:**
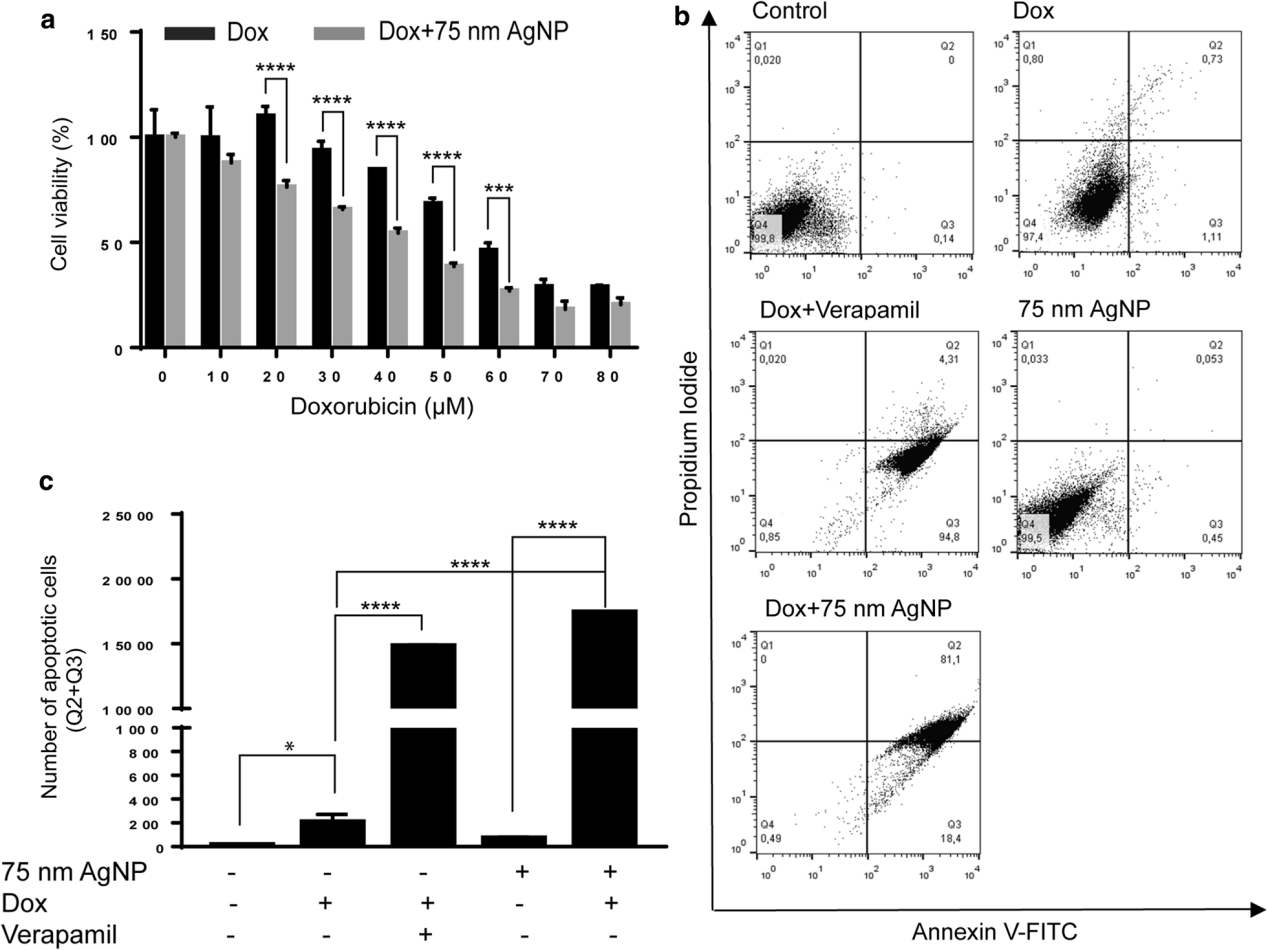
Treatment with 75 nm AgNPs sensitizes drug-resistant MCF-7/KCR cells to doxorubicin-induced apoptosis. **a** Cytotoxicity of doxorubicin and of doxorubicin and 75 nm AgNP combination in MCF-7/KCR cells. **b** Representative dot plots of AnnexinV/PI staining, and **c** number of apoptotic drug-resistant cancer cells following 75 nm AgNP and/or doxorubicin treatment or verapamil administration. The values are the means ± standard deviations of three independent experiments (*P < 0.03 ****P < 0.0001, Fisher’s LSD test)

MCF-7/KCR is a cell line manifesting acquired drug resistance, which was achieved by doxorubicin selection pressure [29, 30]. As 75 nm AgNPs inhibited Pgp efflux activity, we wanted to test whether their administration can sensitize MCF-7/KCR cells to doxorubicin-induced apoptosis. For this, cells were exposed to either doxorubicin alone or to the combination of 75 nm AgNPs and doxorubicin, and following treatments MTT assays were performed. Results show an increase in doxorubicin cytotoxicity after co-treatments with AgNPs, compared to cells receiving only doxorubicin (Fig. 3a). IC_50_ values indicate that when drug-resistant cells are co-treated with 75 nm AgNPs, lower concentration of doxorubicin is required, whereas a higher dose is necessary to reach 50% inhibition when doxorubicin is applied alone. To examine whether the observed cytotoxicity upon 75 nm AgNP + doxorubicin administrations is the result of apoptosis, MCF-7/KCR cells were subjected to treatments described above. Additionally, drug-resistant cells were exposed to a combination of 4 µM verapamil and 20 µM doxorubicin, to assess the apoptotic effect of doxorubicin in MCF-7/KCR cells, where Pgp is blocked with verapamil. AnnexinV-FITC/PI double staining was performed followed by flow cytometry. The representative dot plots (Fig. 3b) and the calculated apoptotic cell numbers (Fig. 3c) indicated that doxorubicin treatment induced apoptosis, which was further enhanced when Pgp activity was blocked by verapamil co-treatments. Single 75 nm AgNP administrations proved to be non-cytotoxic, however, the percentage of double positive cells increased significantly, thus massive apoptosis was triggered when AgNPs and doxorubicin were applied in combination (Fig. 3b, c). These results verify that 75 nm AgNPs sensitize drug-resistant MCF-7/KCR cells to doxorubicin-induced apoptosis.

### AgNPs induce oxidative stress and mitochondrial damage

**Fig. 4:**
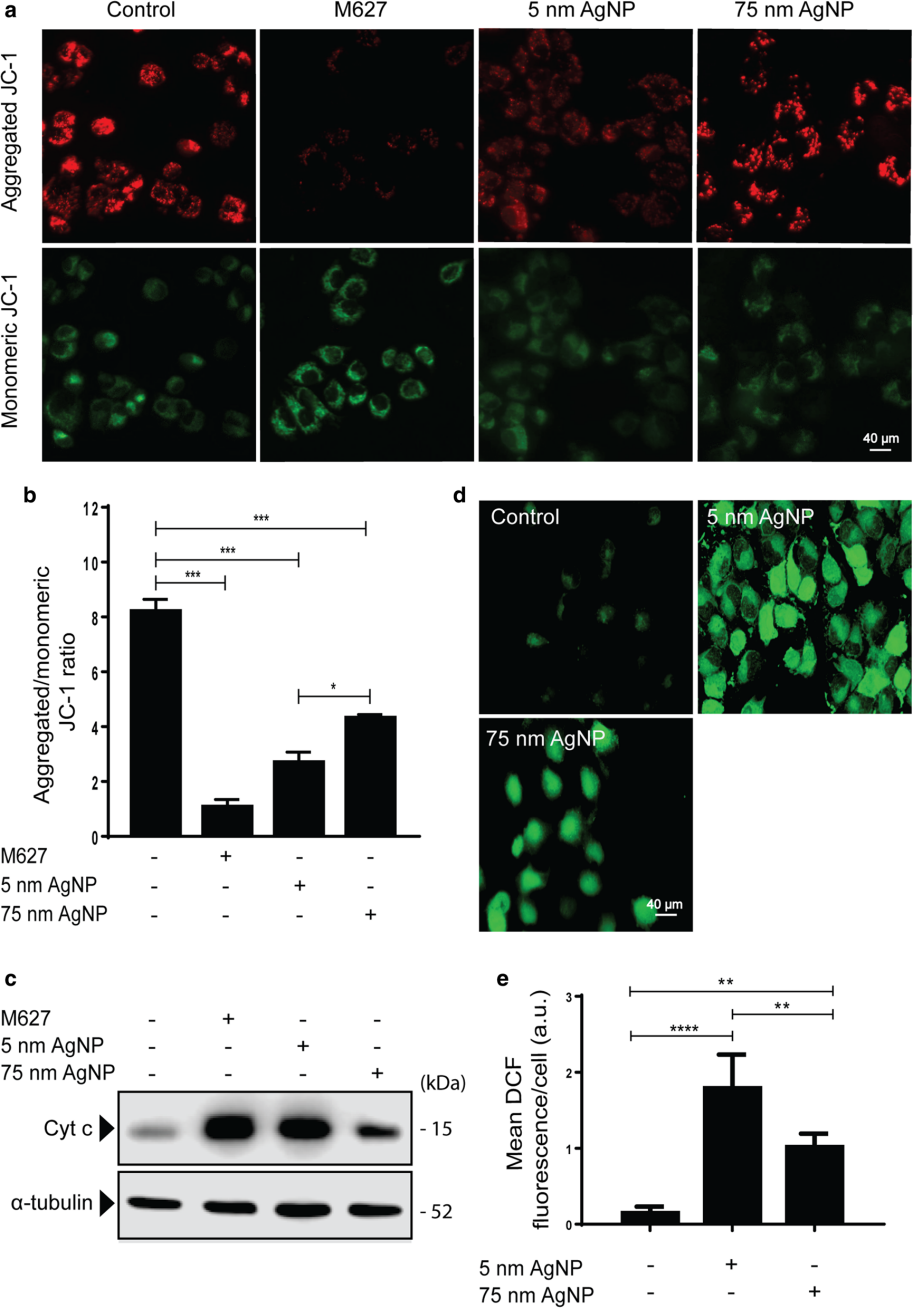
AgNPs induce oxidative stress and mitochondrial damage in MCF-7/KCR cells. **a** Representative images of 5 nm AgNP-, 75 nm AgNP-, or the apoptosis inducer M627-treated MCF-7/KCR cells after JC-1 staining. JC-1 aggregates show red and JC-1 monomers green fluorescence. **b** Aggregated-to-monomeric JC-1 ratio (red-to-green fluorescence ratio) was determined by image analysis. **c** Western blot of cytoplasmic cytochrome c in MCF-7/KCR cells after 5 nm, 75 nm AgNP or M627 treatments. **d** Fluorescence microscopic images of DCFDA-stained, AgNP-treated MCF-7/KCR cells. **e** Mean DCF fluorescence intensity determined by image analysis. Values represent the mean ± standard deviation calculated from 25 cells from two independent experiments (*P < 0.03 **P < 0.002 ***P < 0.0002 ****P < 0.0001, Fisher’s LSD test)

Mitochondrial damage can result in lowered cellular ATP levels, which may ultimately lead to compromised export of Pgp substrates in drug-resistant cells. To examine whether the functional integrity of mitochondria is maintained upon AgNP treatments, we performed JC-1 staining. MCF-7/KCR cells were treated with 5 nm or 75 nm AgNPs or with apoptosis inducer M627. Before JC-1 loading, cells were pre-treated with verapamil for an hour, since we found that JC-1 is a substrate of Pgp. Treatments with M627 decreased the amount of JC-1 aggregates (red fluorescence), whereas increased the quantity of JC-1 monomers (green fluorescence) compared to control cells, indicating significant mitochondrial damage in M627-exposed MCF-7/KCR cells (Fig. 4a, b). Both AgNP treatments caused a decrease in JC-1 aggregates compared to untreated cells, thus reduced red-to-green fluorescence ratio (Fig. 4a, b). It is noteworthy, that 5 nm AgNPs were significantly more detrimental to mitochondria than 75 nm counterparts. Both AgNP treatments triggered the release of cytochrome c into the cytoplasm of MCF-7/KCR cells, however, the effect of 5 nm AgNP exposure was more pronounced, similar to that of M627 (Fig. 4c). Since mitochondrial dysfunction is coupled to oxidative stress, we investigated the ROS generating potential of AgNPs. MCF-7/KCR cells were treated with 5 nm and 75 nm AgNPs and stained with DCFDA. Representative images and mean fluorescence intensity values show that both sized AgNPs induce significant ROS production compared to untreated control, however, 5 nm AgNPs are more potent in this respect than 75 nm AgNPs (Fig. 4d, e). Hence, the mitochondrial damage caused by 75 nm AgNPs is not the basis for its Pgp inhibitory action in MCF-7/KCR cells.

We measured the intracellular silver amount of MCF-7/KCR cells treated with either 5 nm or 75 nm AgNPs by inductively coupled plasma mass spectrometry. Our data indicates that treatments with 5 nm AgNPs result in significantly higher intracellular silver concentrations compared to 75 nm AgNP exposures (Additional file 3). As 75 nm AgNPs, but not 5 nm AgNPs, inhibited greatly the membrane efflux activity, therefore, the observed intracellular silver concentration cannot explain the 75 nm AgNP-induced Pgp inhibition and the associated molecular phenomena in drug-resistant breast cancer cells.

### 75 nm AgNP treatments cause depletion of ER calcium stores and ER stress

**Fig. 5:**
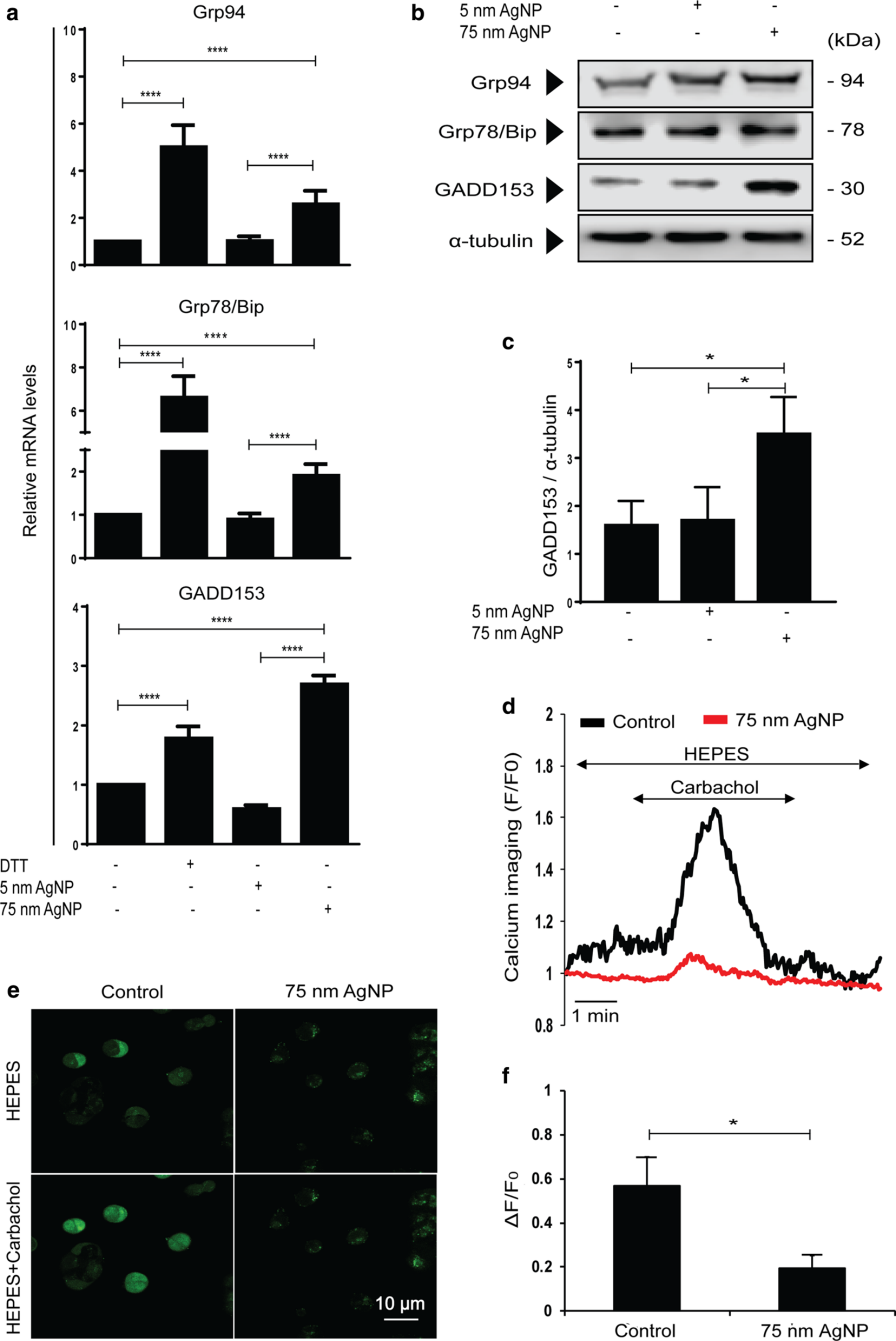
75 nm AgNP treatment depletes ER calcium stores and leads to ER stress. **a** Relative mRNA levels of ER stress markers in MCF-7/KCR cells treated with 75 nm AgNPs. **b** Protein levels of ER stress markers detected by immunoblot. **c** Densitometric quantitation of GADD153 protein levels. **d** Histogram of real-time calcium imaging from at least 5 ROIs (Region of interest) and **e** fluorescent calcium imaging of untreated and 75 nm AgNPs-treated MCF-7/KCR cells upon carbachol administration. Pictures were taken before and 1 min after carbachol exposure. **f** Representative bar graph of cytoplasmic calcium released on carbachol exposure. The values represent the mean ± standard deviation calculated from three independent experiments (*P < 0.03 ****P < 0.0001, Fisher’s LSD test)

Next, we examined whether AgNPs can induce ER stress and activate unfolded protein response (UPR) in drug-resistant cancer cells. Therefore, MCF-7/KCR cells were treated with 5 nm or 75 nm AgNPs or received 2 mM of ER stress-inducing dithiothreitol (DTT) as a positive control. Then mRNA expression levels of various ER stress markers were assessed by RT-qPCR. 75 nm AgNP treatment induced the expression of ER chaperons Grp94 and Grp78/Bip, as well as of the ER stress-provoked pro-apoptotic mediator GADD153 in MCF-7/KCR cells (Fig. 5a). Importantly, 5 nm AgNPs did not trigger expressional changes in ER stress markers in MCF-7/KCR cells. GADD153 protein expression was elevated following 75 nm AgNPs treatment (Fig. 5b, c), whereas Grp78 and Grp94 protein levels remained unchanged upon 75 nm AgNP expositions (Fig. 5b). This result is not surprising, as many cancer cell types, including MCF-7, express high basal levels of GRP94 and GRP78 [34], hence further increases in their protein levels are not always possible during ER stress.

Frequently, depletion of ER calcium stores is the reason behind ER stress [35], therefore we measured the calcium flux related to 75 nm AgNP treatment in drug-resistant cancer cells. Control and 75 nm AgNP-treated MCF-7/KCR cells were exposed to 100 µM carbachol, an agent stimulating IP_3_-receptors to release ER-stored calcium (Fig. 5d). The released ER calcium is immediately sensed by the pre-loaded fluorescent dye Fluo-4, manifesting an increased fluorescence and enabling a rapid, real-time detection of calcium flux from pre-set ROIs (Fig. 5e). As Fluo-4 is a substrate of Pgp, we pre-treated the samples with the Pgp inhibitor Quinidine (this time not with verapamil, as it can influence calcium flux) before measurements. If AgNP treatment leads to ER calcium depletion, an exposure to carbachol would result little or no calcium release into the cytoplasm. In fact, fluorescent microscopic images and the associated histogram prove that MCF-7/KCR cells treated with 75 nm AgNPs failed to respond to carbachol, indicating that ER calcium stores are already depleted or are below the detectable concentration in Pgp-overexpressing drug-resistant cancer cells (Fig. 5d–f).

## 75 nm AgNPs disrupt cellular Pgp distribution

**Fig. 6:**
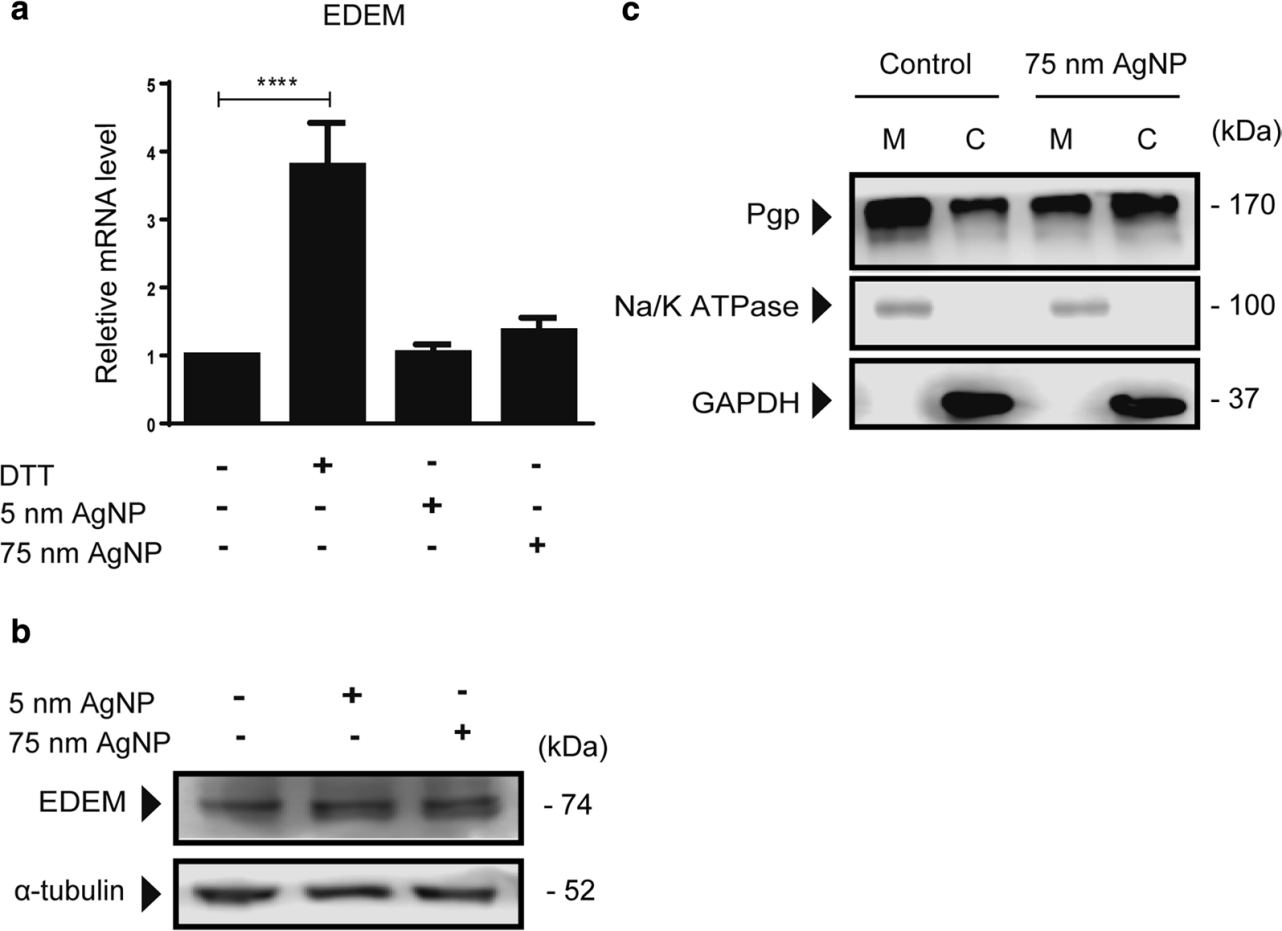
75 nm AgNP treatment disrupts Pgp protein distribution between the plasma membrane and cytoplasm of MCF-7/KCR cells. **a** Relative mRNA and **b** protein levels of EDEM, a misfolded glycoprotein-binding protein in MCF-7/KCR cells treated with AgNPs. **c** Pgp protein levels determined from the plasma membrane (Na^+^/K^+^ ATPase-positive) and cytoplasmic fractions (GAPDH-positive) of control and 75 nm AgNP treated MCF-7/KCR cells (M-Plasma membrane; C-Cytoplasm)

Activation of ER stress and unfolded protein response increase the amount of unfolded/misfolded proteins, which are ultimately directed to ER-associated protein degradation (ERAD) by activated ER degradation-enhancing α-mannosidase-like protein (EDEM) [36]. However, in cases of critical ER calcium levels, unfolded proteins do not enter the degradation machinery but accumulate intracellularly. We presumed that 75 nm AgNP treatments lead to accumulation of misfolded/unfolded Pgp in the ER, cytoplasm or both, hence the number of Pgp on the plasma membrane decreases. To prove this hypothesis, MCF-7/KCR cells were treated with 5 nm or 75 nm AgNPs, or with ER stress-inducing DTT, and the transcriptional and translational activation of EDEM was examined by RT-qPCR and western blot, respectively. Our results indicated that both AgNPs failed to induce EDEM expression (Fig. 6a, b), suggesting that ERAD has not been activated. To strengthen this argument, we treated MCF-7/KCR cells with 75 nm AgNPs and determined the Pgp protein distribution between plasma membrane and cytoplasm. Purity of plasma membrane and cytoplasmic fractions were verified by detection of Na^+^/K^+^ ATPase and GAPDH, respectively. Our results indicated that treatment with 75 nm AgNPs lowered the amount of Pgp in the plasma membrane (M), with a collateral increase in the cytoplasmic fraction (C) compared to the untreated control (Fig. 6c). This shift in Pgp distribution explains the observed inhibition of Pgp transporter activity without downregulating its expression by 75 nm AgNPs.

## Discussion

P-glycoprotein (Pgp) is a plasma membrane localized ABC transporter, related inherently to the development of MDR cancer. Even though the molecular events behind the overexpression and elevated efflux activity of Pgp have been intensively studied, we still not own proper pharmaceutical strategies to defeat intrinsic or acquired resistant cancer phenotypes, leading to lower survival rates of patients subjected to conventional chemotherapy [37]. Pgp grants drug-resistance to cancer cells by an efflux mechanism powered by ATP hydrolysis, where structurally and functionally unrelated drugs are expelled from cancer cells [6]. Breast cancer might develop MDR associated with Pgp overexpression [5], which is a major reason for chemotherapy failure and cancer recurrence. Hence, inhibiting Pgp activity could be a logical strategy to improve the efficacy of breast cancer therapy.

Silver nanoparticles have recently gained interest in nanomedicine due to unique biological properties, which are largely dependent on nanoparticle size [38–44]. Our group has previously reported that AgNPs of 28 nm inhibited Pgp expression and function and sensitized multidrug-resistant colon adenocarcinoma cells to various antineoplastic agents [27]. Along this line, the present study aimed to elucidate (i) whether AgNPs are capable to attenuate acquired MDR by modulating Pgp activity; (ii) if yes, is Pgp inhibition AgNP size-dependent; iii, finally, what is the mechanism behind size-dependent Pgp inhibition?

To answer these questions, AgNPs of two different sizes were synthetized [31] and multidrug-resistant MCF-7/KCR cells, overexpressing Pgp and exhibiting active efflux activity, were developed from MCF-7 cells using doxorubicin selection. AgNPs effectively killed MCF-7/KCR cells, but as expected, these cells were more resistant to the cytotoxic effects of both 5 nm and 75 nm AgNPs compared to MCF-7 cells, possibly due to their more efficient cellular stress management mechanisms. In accord with previous reports [41–44], AgNP cytotoxicity depended on nanoparticle size, as 5 nm AgNPs were more toxic than 75 nm AgNPs.

Interestingly, not only cytotoxic propensity, but the inhibition of Pgp efflux activity proved to be dependent on nanoparticle size, since 75 nm AgNPs, but not 5 nm counterparts, were capable to reduce Pgp transport activity in drug-resistant MCF-7/KCR cells, although no change in Pgp protein expression was observed (Fig. 2). This stresses on the point that for efficient modulation of Pgp action, nanoparticles with the proper size must be selected. In fact, using the appropriate AgNPs, reversal of drug resistance might be achieved and cancer cells could be sensitized to chemotherapeutic drugs. Hence, we proved that the cytotoxic and apoptosis-inducing potency of doxorubicin, a Pgp substrate and a first line chemotherapeutic drug to treat breast cancers, has been significantly raised in co-treatments with 75 nm AgNPs in multidrug-resistant MCF-7/KCR cells.

Inhibition of Pgp efflux activity by 75 nm AgNP treatment, without compromising the Pgp expression was quite intriguing, which prompted us to investigate the underlying cellular mechanisms. Initially we hypothesized, that augmented ROS production upon AgNP treatments [38–44] would ultimately influence drug transport activity by causing mitochondrial dysfunction [44–46], diminishing mainly mitochondrium-derived ATP levels and making it insufficient to fuel Pgp efflux. In fact, in a recent report, Pgp inhibitory potential of the novel compound RY10-4 was partially attributed to its cellular ATP diminishing capacity [47]. However, our results indicated that 75 nm AgNPs were less potent than 5 nm AgNPs in inducing ROS generation and damaging mitochondria (Fig. 4). Therefore, oxidative stress-related mitochondrial dysfunction is not the reason behind reduced Pgp activity in 75 nm AgNP-treated drug-resistant MCF-7/KCR cells. Furthermore, since we found higher intracellular silver concentration in 5 nm AgNP-treated drug-resistant cells than in 75 nm AgNP-exposed counterparts (Additional file 3), we concluded that although high amounts of reactive silver might influence cytotoxicity, this is not the feature which directs the observed inhibitory effects of 75 nm AgNPs on P-glycoprotein. We believe that the 75 nm AgNP-induced Pgp inhibition and the associated molecular phenomena in drug-resistant breast cancer cells must be related rather to AgNP size.

Endoplasmic reticulum is a major assembly site of secretory and integral membrane proteins. The ER lumen is rich in calcium, which is essential for continuous functioning of ER protein quality control mechanisms, like the calnexin/calreticulin cycle. Calnexin and calreticulin are ER resident lectins that ensure proper folding and oligomerization of glycoproteins in the ER [48–50]. ER stress disturbs the homeostasis of protein folding machinery and is manifested by activation of ER stress response elements and ER-associated degradation (ERAD) [36, 49, 50]. It has been reported previously that AgNPs are able to induce ER stress and disturb cellular calcium homeostasis [51, 52]. Hence, we hypothesized that 75 nm AgNPs, by inducing ER stress in drug-resistant cells, decrease the number of properly folded Pgp reaching the plasma membrane, where these transporters should manifest their function. Our results showed that 75 nm AgNPs, but not 5 nm AgNPs induce the expression of ER stress markers (Grp94, Grp78/Bip, GADD153) in MCF-7/KCR cells. Finally, we proved that treatment of drug-resistant cells with 75 nm AgNPs depletes ER calcium levels (Fig. 5), which is the probable reason for ER stress induction. However, it is yet to be verified whether ER calcium loss is leading to ER stress or ER stress (due to other factors) is leading to calcium loss.

Under ER stress the amount of unfolded/misfolded proteins increases, which molecules are ultimately degraded by ERAD. For this, EDEM should be transcriptionally and translationally activated, in order to direct terminally misfolded glycoproteins from futile calnexin/calreticulin cycles to ERAD [35, 36]. Under such circumstances a reduction in the total Pgp levels is expected. Although we have verified the induction of ER stress, we have not observed a decrease in Pgp protein levels in MCF-7/KCR cells treated with 75 nm AgNPs (Fig. 2). This can be explained by critically low ER calcium levels, because in such cases terminally misfolded glycoproteins do not or just slowly enter ERAD, and the activation of EDEM is not observable. In fact, we showed that EDEM expression levels were not changed upon 5 nm and 75 nm AgNPs treatments (Fig. 6), which can explain why under ER stress conditions, induced by 75 nm AgNPs, the Pgp transporter activity was inhibited, but this glycoprotein was not subjected to ERAD, possibly due to critically depleted ER calcium levels. The validity of this hypothesis was further supported by a shift in the distribution of Pgp between plasma membrane and cytoplasm upon 75 nm AgNP treatment. Our results demonstrated that plasma membrane Pgp levels significantly decreased, whereas cytoplasmic Pgp levels increased in MCF-7/KCR cells treated with 75 nm AgNPs compared to untreated control (Fig. 6).

## Conclusions

Our study revealed that larger sized AgNPs are potent tools for modulating Pgp activity and sensitizing multidrug resistant breast cancers to anticancer agents. We provide evidence that exploitation of ER stress can be a propitious target in defeating multidrug resistance in cancers. This is a highly relevant finding as it renders AgNPs attractive candidates in rational design of therapeutically useful agents for tumor targeting.

## Additional files

**Additional file 1.** Fluorescence microscopic images of drug-resistant MCF-7/KCR cells loaded with JC-1 dye without and with Verapamil pre-treatment. Images indicate that verapamil treatment improved the retention of JC-1 dye in drug-resistant cancer cells. (JC-1 staining procedure is described in the Materials and Methods section).

**Additional file 2.** Internalization of AgNPs in drug-resistant MCF-7/KCR cells. Intracellular AgNPs were visualized by transmission electron microscopy. Representative TEM images of 5 nm and 75 nm AgNP-treated MCF-7/KCR cells and the enlarged sections verify the presence of AgNP aggregates inside cells (black arrow heads). n and c indicate nucleus and cytoplasm, respectively. For TEM imaging of biological samples 10^5^ cells were seeded onto 0.4 µm pore sized polyester membrane inserts (Corning) placed in a 6-well plate. Cells were allowed to grow until the following day when they were treated with AgNPs for 24 h. Then cells were washed and fixed in 4% glutaraldehyde in PBS and embedded in gelatine. The obtained specimens were sliced to 1–2 mm cubes, which were embedded in epoxy (Epon 812, EMS) by a routine TEM sample preparation protocol. Blocks were trimmed, thin sections of 70 nm were obtained and stained with uranyl and lead solutions. Images were captured by a Philips CM10 electron microscope using 100 kV voltage. TEM micrographs were taken by a Megaview G2 digital camera (ITEM, Olympus).

**Additional file 3.** Intracellular silver concentrations of MCF-7/KCR cells treated with either 5 nm or 75 nm AgNPs determined by inductively coupled plasma mass spectrometry (ICP-MS). Results indicate that treatments with 5 nm AgNPs lead to significantly higher intracellular silver concentrations compared to 75 nm AgNP exposures. The values represent the mean ± standard deviation calculated from three independent experiments (***, P<0.0002 ****, P <0.0001, Fisher’s LSD test). To determine the intracellular silver amount of AgNP-treated as well as of control MCF-7/KCR cells by ICP-MS (Quadrupole Agilent 7700x SP-ICP-MS), cells were digested with cc HCl for 90 min at 90°C, then an equal volume of cc HNO_3_ was added and the samples were further digested for another 90 min. The resulting liquid was filtered on 0.45 nm hydrophilic membrane filter and diluted to 100 mL final volume.

## Abbreviations

AgNPs: silver nanoparticles
ER: endoplasmic reticulum
ERAD: endoplasmic reticulum-associated degradation
FITC: fluorescein isothiocyanate
MDR: multidrug resistance
Pgp: P-glycoprotein
PI: propidium Iodide
RH123: rhodamine 123
ROS: reactive oxygen species
