# Supplementary figures and images for: Endoplasmic reticulum stress: major player in size-dependent inhibition of P-glycoprotein by silver nanoparticles in multidrug-resistant breast cancer cells
Source: J Nanobiotechnology. 2019 Jan 22;17:9. doi: 10.1186/s12951-019-0448-4 (PMC6341731; doi:10.1186/s12951-019-0448-4)

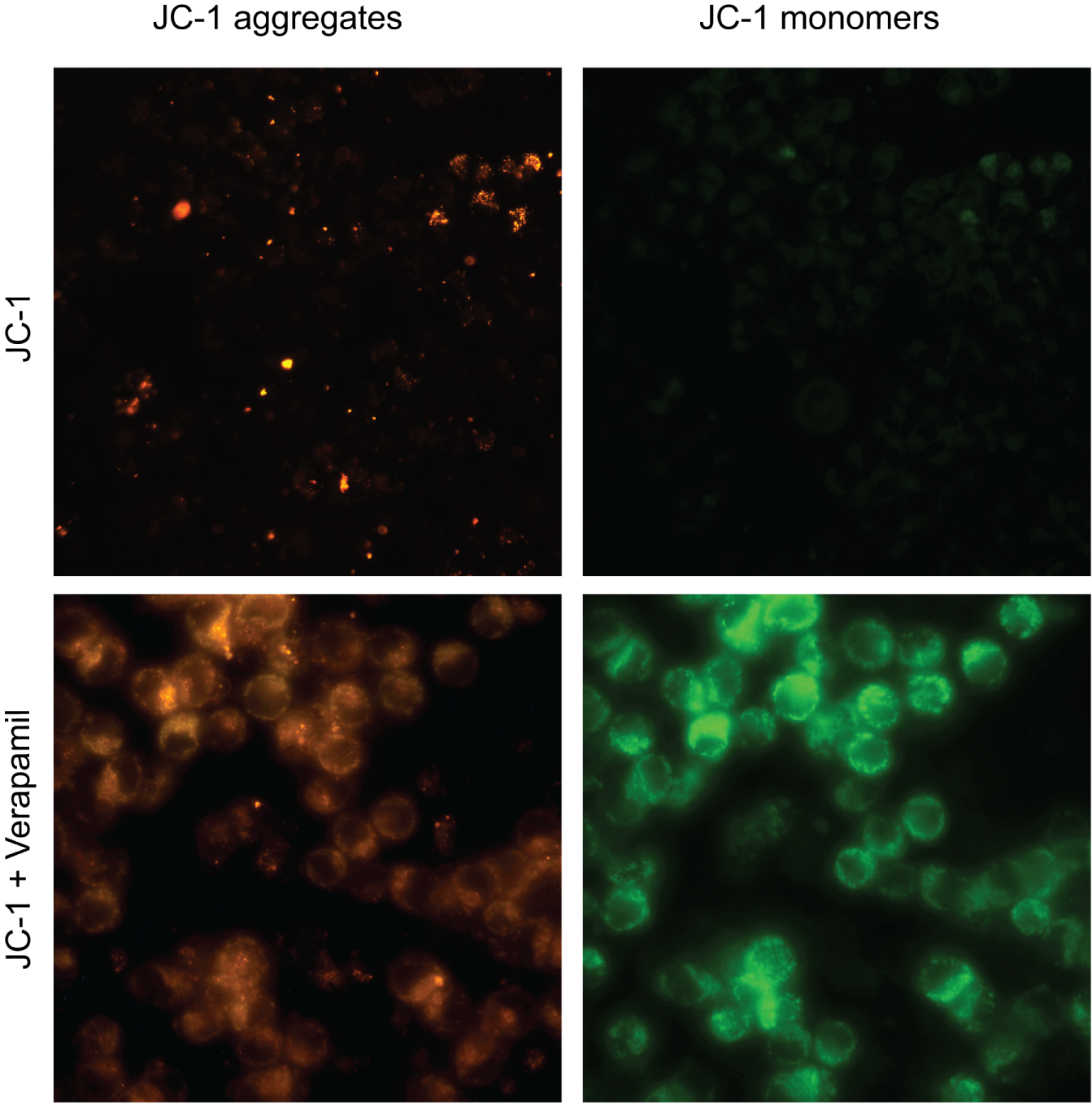

Supplement: Supplementary file 1 — Additional file 1. Fluorescence microscopic images of drug-resistant MCF-7/KCR cells loaded with JC-1 dye without and with Verapamil pre-treatment. Images indicate that verapamil treatment improved the retention of JC-1 dye in drug-resistant cancer cells. (JC-1 staining procedure is described in the Materials and Methods section). [file 12951_2019_448_MOESM1_ESM.tif]

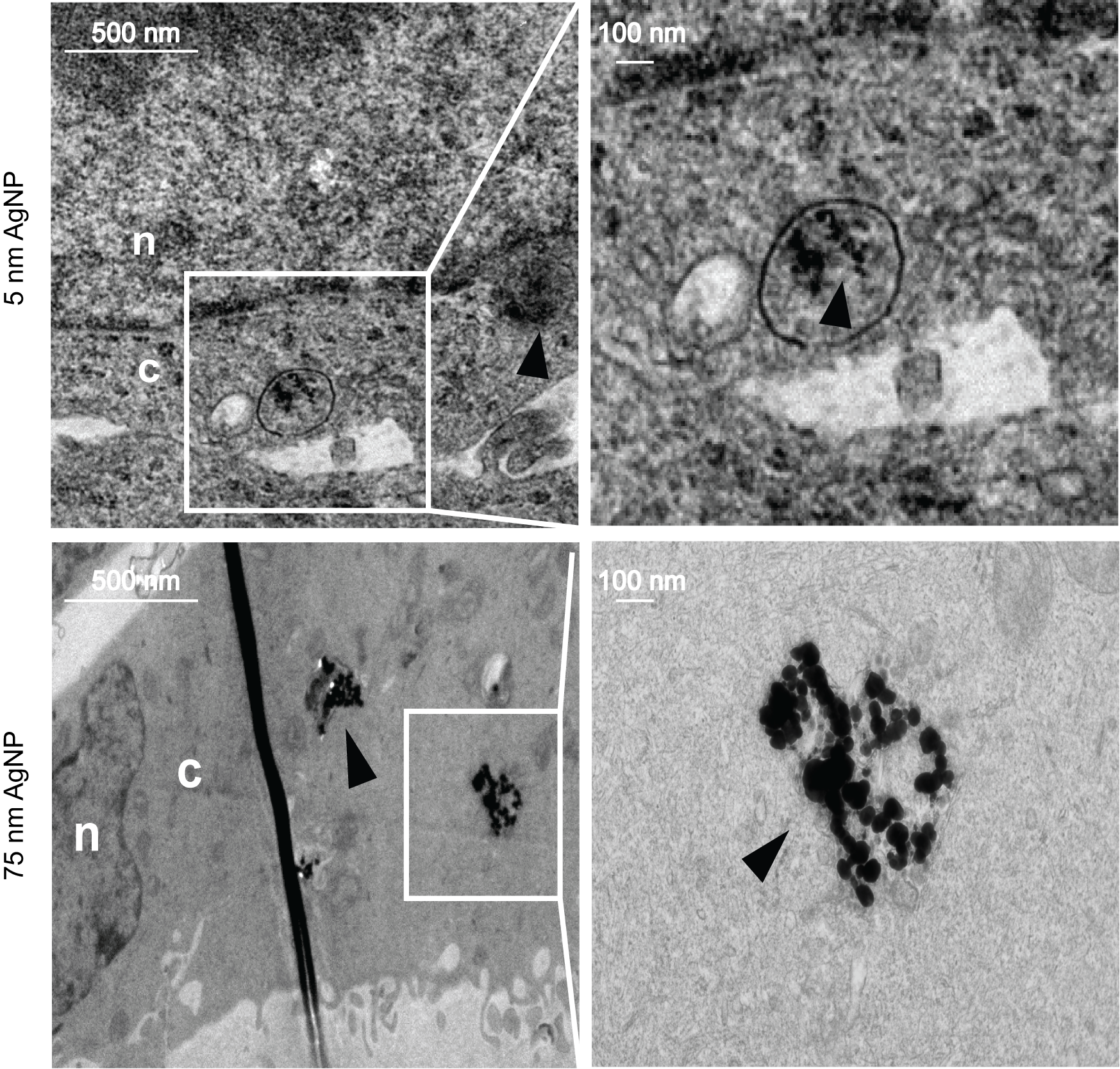

Supplement: Supplementary file 2 — Additional file 2. Internalization of AgNPs in drug-resistant MCF-7/KCR cells. Intracellular AgNPs were visualized by transmission electron microscopy. Representative TEM images of 5 nm and 75 nm AgNP-treated MCF-7/KCR cells and the enlarged sections verify the presence of AgNP aggregates inside cells (black arrow heads). n and c indicate nucleus and cytoplasm, respectively. For TEM imaging of biological samples 105 cells were seeded onto 0.4 µm pore sized polyester membrane inserts (Corning) placed in a 6-well plate. Cells were allowed to grow until the following day when they were treated with AgNPs for 24 h. Then cells were washed and fixed in 4% glutaraldehyde in PBS and embedded in gelatine. The obtained specimens were sliced to 1–2 mm cubes, which were embedded in epoxy (Epon 812, EMS) by a routine TEM sample preparation protocol. Blocks were trimmed, thin sections of 70 nm were obtained and stained with uranyl and lead solutions. Images were captured by a Philips CM10 electron microscope using 100 kV voltage. TEM micrographs were taken by a Megaview G2 digital camera (ITEM, Olympus). [file 12951_2019_448_MOESM2_ESM.tif]

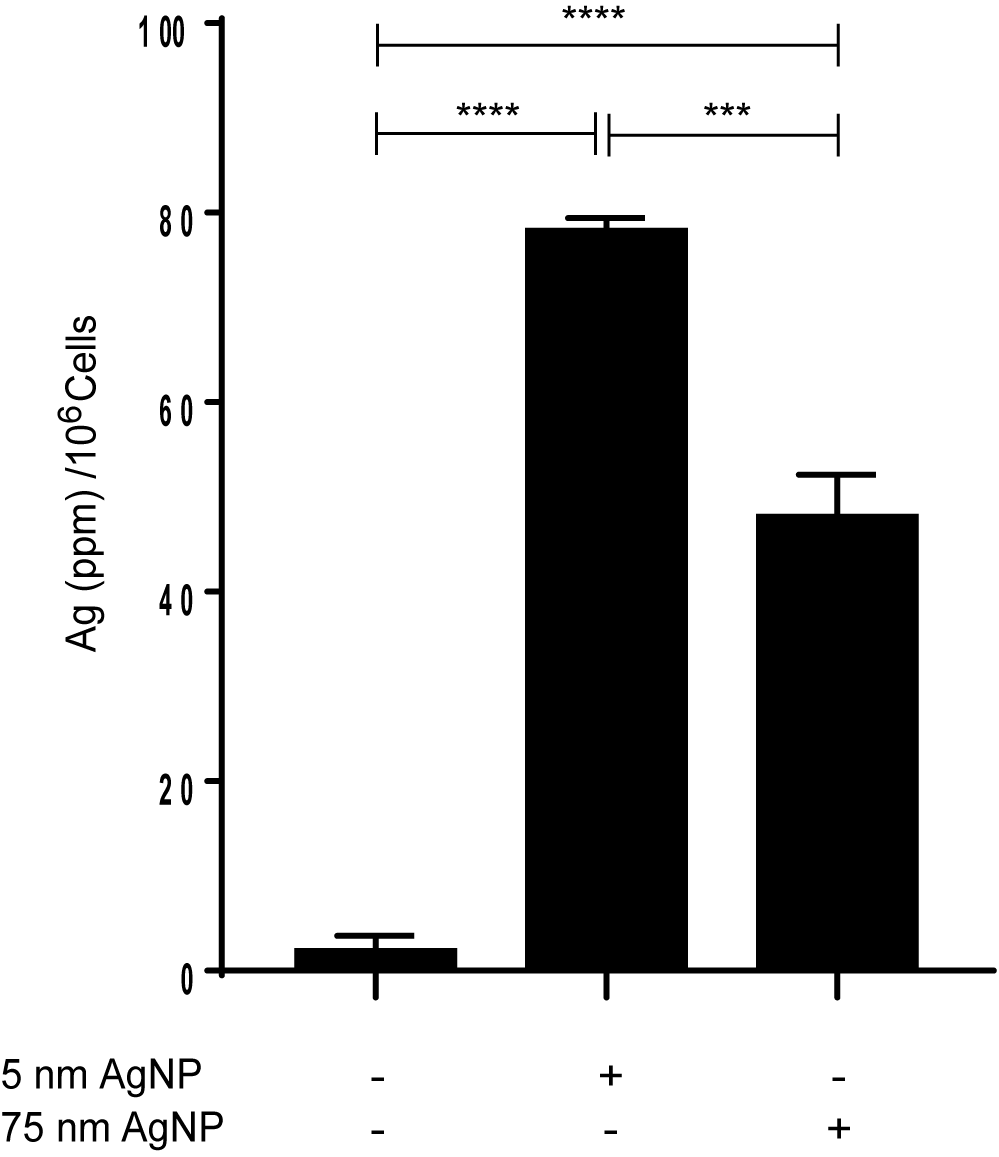

Supplement: Supplementary file 3 — Additional file 3. Intracellular silver concentrations of MCF-7/KCR cells treated with either 5 nm or 75 nm AgNPs determined by inductively coupled plasma mass spectrometry (ICP-MS). Results indicate that treatments with 5 nm AgNPs lead to significantly higher intracellular silver concentrations compared to 75 nm AgNP exposures. The values represent the mean ± standard deviation calculated from three independent experiments (***, P<0.0002 ****, P <0.0001, Fisher’s LSD test). To determine the intracellular silver amount of AgNP-treated as well as of control MCF-7/KCR cells by ICP-MS (Quadrupole Agilent 7700x SP-ICP-MS), cells were digested with cc HCl for 90 min at 90°C, then an equal volume of cc HNO3 was added and the samples were further digested for another 90 min. The resulting liquid was filtered on 0.45 nm hydrophilic membrane filter and diluted to 100 mL final volume. [file 12951_2019_448_MOESM3_ESM.tif]
